# Risk Factors for Autism Spectrum Disorder in Individuals Born Preterm: A Systematic Review and Meta-Analysis of Population-Based Studies

**DOI:** 10.1016/j.bpsgos.2025.100535

**Published:** 2025-05-14

**Authors:** Bo Yang, Nina Zaks, Eero Kajantie, Monica S.M. Persson, Abraham Reichenberg, Mika Gissler, Kari Risnes, Alexander Kolevzon, Ulrika Ådén, Ezra Susser, Martina Persson, Jonas F. Ludvigsson, Kristiina Tammimies, Liona C. Poon, Benjamin Yip, Nora Döring, Sven Sandin, Weiyao Yin

**Affiliations:** a JC School of Public Health and Primary Care, The Chinese University of Hong Kong, Hong Kong, China; b Department of Child and Adolescent Psychiatry, NYU Langone Health, New York, New York; c Finnish Institute for Health and Welfare, Helsinki, Finland; d Clinical Medicine Research Unit, MRC Oulu, Oulu University Hospital and University of Oulu, Oulu, Finland; e Department of Clinical and Molecular Medicine, Norwegian University of Science and Technology, Trondheim, Norway; f Children’s Hospital, Helsinki University Hospital and University of Helsinki, Helsinki, Finland; g Department of Medical Epidemiology and Biostatistics, Karolinska Institutet, Stockholm, Sweden; h Department of Psychiatry, Icahn School of Medicine at Mount Sinai, New York, New York; i Seaver Autism Center for Research and Treatment, Icahn School of Medicine at Mount Sinai, New York, New York; j Department of Data and Analysis, Finnish Institute for Health and Welfare, Helsinki, Finland; k Department of Molecular Medicine and Surgery, Karolinska Institute, Stockholm, Sweden; l Academic Primary Health Care Centre, Stockholm, Sweden; m Department of Clinical and Molecular Medicine, Norwegian University of Science and Technology, Trondheim, Norway; n Centre for Health Care Improvement, St. Olavs Hospital, Trondheim, Norway; o Children’s Clinic, St. Olavs Hospital, Trondheim, Norway; p Department of Pediatrics, Icahn School of Medicine at Mount Sinai, New York, New York; q Department of Bioclinical Sciences, Linköping University, Linköping, Sweden; r Department of Women’s and Children’s Health, Karolinska Institutet, Stockholm, Sweden; s Department of Epidemiology, Mailman School of Public Health, Columbia University, New York, New York; t New York State Psychiatric Institute, New York, New York; u Department of Clinical Science and Education, Karolinska Institutet, Stockholm, Sweden; v Department of Paediatrics, Diabetes/Endocrinology, Sachsska Childrens and Youth Hospital, Stockholm, Sweden; w Department of Paediatrics, Örebro University Hospital, Örebro, Sweden; x Department of Medicine, Columbia University College of Physicians and Surgeons, New York, New York; y Center of Neurodevelopmental Disorders (KIND), Centre for Psychiatry Research, Department of Women’s and Children’s Health, Karolinska Institutet, Stockholm, Sweden; z Astrid Lindgren Children’s Hospital, Karolinska University Hospital, Region Stockholm, Stockholm, Sweden; aa Department of Obstetrics and Gynaecology, The Chinese University of Hong Kong, Hong Kong, China

**Keywords:** ASD, Autism spectrum disorder, Meta-analysis, Population-based, Preterm birth, Systematic review

## Abstract

**Background:**

Preterm children are at an increased risk of autism spectrum disorder (ASD), although the determinants of ASD among them remain unclear. In this systematic review and meta-analysis, we summarize the population-based literature on ASD risk factors in preterm-born individuals.

**Methods:**

We searched Ovid MEDLINE, Embase, and Web of Science through September 2023 for population-based studies on ASD risk factors in preterm cohorts (<37 weeks’ gestation). From 3921 articles, 19 met inclusion criteria. Registered in PROSPERO and following Preferred Reporting Items for Systematic Reviews and Meta-Analyses (PRISMA) guidelines, data were extracted and analyzed using fixed and random effects meta-analysis models. Primary outcomes included ASD risk factors, pooled when consistently examined in at least 2 studies.

**Results:**

The qualitative synthesis included 16 cohort studies, 2 case-control studies, and 1 cross-sectional study, while 3 cohort studies were included in the meta-analysis. Sample sizes ranged from 410 to 515,789. Male sex was the only risk factor eligible for meta-analysis and was associated with increased risk of ASD (relative risk 3.04; 95% CI, 2.02–4.57). Low birth weight suggested a potential positive association with ASD, while neonatal jaundice showed no clear link. Pooled estimates were unavailable for these exposures due to heterogeneity in exposure definitions and effect measures. All other risk factors were examined in two or fewer studies.

**Conclusions:**

Significant knowledge gaps remain regarding the risk of ASD in individuals born preterm. The only consistent risk factor identified is male sex, with potential links to low birth weight. To better understand the differences in ASD etiology between preterm and term-born individuals, further research is crucial.

## Plain Language Summary

Premature birth is a recognized risk factor for autism spectrum disorder (ASD). While the underlying causes of ASD may differ between preterm and full-term children, there is a significant gap in our understanding of ASD risk specifically within the preterm population. Therefore, we conducted a systematic review of all population-based studies examining risk factors for ASD in preterm-born cohorts. Besides sex, our findings revealed limited available evidence, which hinders a comprehensive comparison of ASD risk between preterm and full-term children.

Autism spectrum disorder (ASD) is a neurodevelopmental disorder with an estimated heritability exceeding 80% (1,2). While genetic propensity explains most of an individual’s risk, other factors may interact with gene variations to contribute to the disorder (3,4). One such factor is preterm birth (birth before 37 completed weeks of gestation) (5). Investigations have shown more than a 3-fold increased risk of ASD in individuals who were born preterm compared to the general population (6). Furthermore, the risk of ASD increases with lower gestational age (GA), reaching 4-fold levels in children born at week 24.

**Figure 1:**
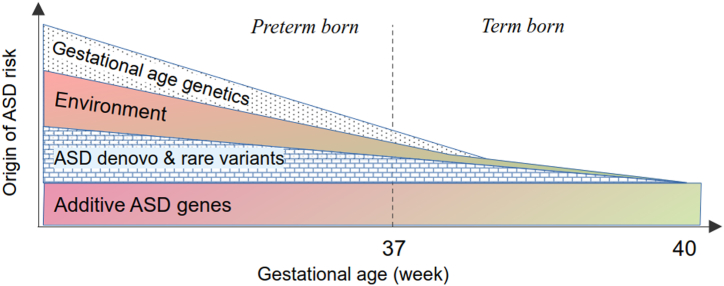
Mechanisms/sources of risk linking gestational age to autism spectrum disorder (ASD).

The etiology of ASD is driven by multiple mechanisms and sources of risk (Figure 1). These can be related to 1) the genetic determinants of GA, which may directly or indirectly affect ASD risk; 2) environmental influences that act on both GA and ASD, such as maternal smoking (7,8), diabetes (9,10), hypertensive disorders (2,11), obesity (3,5,12), and intrauterine infections (13,14); and 3) de novo and rare genetic variants that are more prevalent at earlier GAs, which may result in malformations and genetic disorders that are linked with ASD (15, 16, 17, 18).

Therefore, the risk architecture for ASD may differ between individuals who were born preterm and individuals who were born at term, including the magnitude of risk associated with each risk factor, the underlying genetics (19), and the gene-environment interactions that contribute to the disorder. Given the improved survival of infants born preterm and a growing population at risk of ASD, there is cause to investigate the pathways of risk between preterm birth and ASD. Only large, population-based studies of individuals who were born preterm can robustly identify factors that contribute to ASD risk beyond the effects of prematurity itself. Therefore, we conducted this review to assess the population-based literature on risk factors for ASD within cohorts of individuals who were born preterm.

## Methods and Materials

### Study Design

This systematic review and meta-analysis followed the Preferred Reporting Items for Systematic Reviews (PRISMA) guidelines (20) and was registered on PROSPERO (registration number: CRD42020214689) in November 2020. Only population-based studies that examined risk factors for ASD in preterm populations were included, without geographic restrictions. Studies that did not use clinician-based diagnoses of ASD were excluded. Search strategy, data extraction, and study quality assessment can be found in the Supplemental Methods.

### Data Synthesis and Meta-analytic Assessments

We performed meta-analyses on risk factors examined consistently across more than 2 studies. Fixed and random effects models with inverse variance weighting were fitted (21). Pooled estimates of ASD risk (relative risk [RR]) with 2-sided 95% CIs were calculated. Fully adjusted effect estimates from studies were included. When there was substantial heterogeneity (*I*^*2*^ ≥ 50%) (22), random effect estimates were reported. For overlapping study populations, the larger study was included. The meta package (version 6.0-0) and metafor package (version 3.8-1) in R (version 4.1.0) were used. Hypothesis tests were based on a 2-sided 5% significance level.

## Results

**Figure 2:**
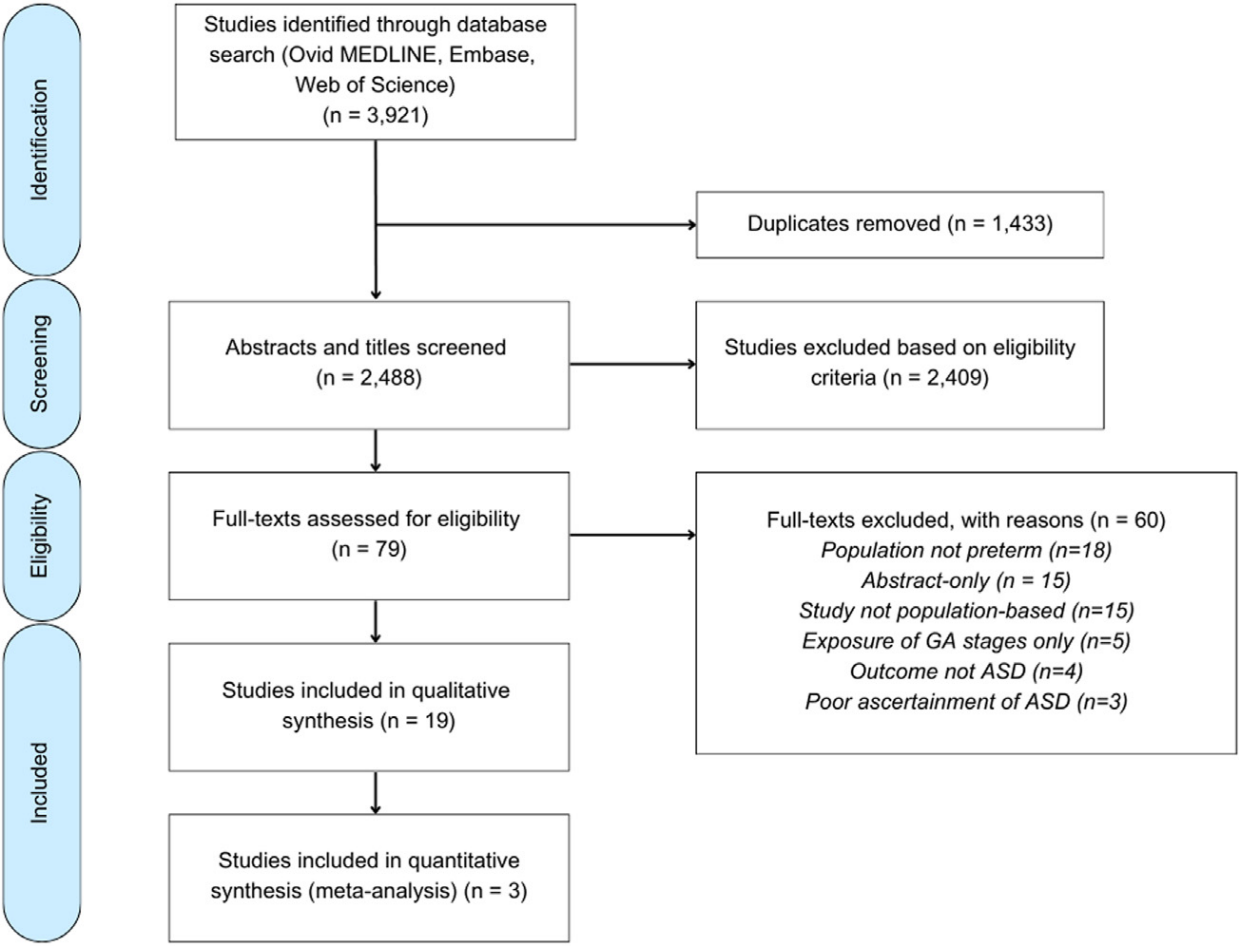
Preferred Reporting Items for Systematic Reviews and Meta-Analyses (PRISMA) flow diagram. ASD, autism spectrum disorder; GA, gestational age.

Of 3921 studies, 1433 were duplicates, and 2409 were excluded based on the title and abstract. Seventy-nine full texts were reviewed, 19 of which were included in the review and 3 of which were included in the meta-analysis (Figure 2).

### Study Characteristics

Of the 19 included studies, 16 had cohort designs. Of these, 8 used the U.S.-based Extremely Low Gestational Age Newborn (ELGAN) Study (23, 24, 25, 26, 27, 28, 29, 30), and 4 used U.S.-based multisite regional health care databases (31, 32, 33, 34). Four studies relied on national health registers, 2 in Taiwan (35,36) and 2 in the Nordic countries (37,38). The 2 case-control studies were derived from the multisite Study to Explore Early Development (SEED) in the United States (39) and the national health registers in Sweden (40). The single cross-sectional study used the National Survey of Children’s Health in the United States (41).

Twelve studies were rated high quality, 6 moderate quality, and 1 low quality according to the Newcastle-Ottawa scale, and the latter study warranted exclusion in the meta-analysis (Table S2). Basic characteristics of the studies are shown in Table S3. The effect estimates of each risk factor are presented in Table 1.

### ASD Ascertainment

Studies of the ELGAN cohort (23, 24, 25, 26, 27, 28, 29, 30) used standardized diagnostic tools (the Autism Diagnostic Interview-Revised [ADI-R] and the Autism Diagnostic Observation Schedule-Second Edition [ADOS-2]) and the Social Communication Questionnaire screening tool. One study relied on parental recall of whether a health care provider had ever informed them that their child had ASD (41). One study used the ADOS and the ADI-R (39), and 2 studies ascertained ASD cases through U.S. state-level governmental services for individuals with developmental disabilities (identified through medical and other reports) (31,34). Seven studies retrieved ASD diagnoses assigned by physicians in medical records (32,33,35, 36, 37, 38,40) (Table S3).

**Table 1:** Summary of Examined Risk Factors for Autism Spectrum Disorder in Individuals Born Preterm

Exposure Category	Study-Defined Exposure	Effect Estimate	Author, Year	No. ASD/No. Preterm
Characteristics at Birth				
Race/Ethnicity	Race/ethnicity	Hispanic: 0.60 (0.28, 1.31) other: 0.62 (0.20, 1.99) Ref. group: White	Bakian, 2018 (31)	112/4855
	Racial identity (White, Black, Hispanic, other)	NS	Joseph, 2017 (23)	59^§^/840
Sex	Child sex (male)	ASD+/ID−: OR 2.1 (0.9, 5.0) ASD+/ID^+^: OR 2.9 (1.3, 6.8)^∗^ ASD−/ID+: OR 2.1 (1.2, 3.6)^∗^	Joseph, 2017 (23)	59^§^/840
	Male	RR 2.81 (1.78, 4.44)^∗^	Bakian, 2018 (31)	112/4855
	Male	OR 3.9 (3.0, 5.1) (crude)^∗^, 4.1 (3.1, 5.3) (adj.)^∗^	Hwang, 2013 (36)	411/30,025
	Male sex	OR 2.0 (1.1, 3.6)^∗^	Kuban, 2016 (25)	61/874
Apgar Score	5-min Apgar score	RR 0.96 (0.86, 1.07)	Bakian, 2018 (31)	112/4855
	Apgar <7 at 5 min	NS	Getahun, 2017 (32)	524/27,949
Birth Weight/Size	Birth weight	<750 g: OR 3.2 (1.5, 7.1) (crude), 3.9 (1.8, 8.8) (adj.)^∗^ 750–1499 g: OR 1.7 (1.3, 2.4) (crude)^∗^, 2.0 (1.5, 2.8) (adj.)^∗^ 1500–2499 g: OR 1.0 (0.8, 1.3) (crude), 1.1 (0.9, 1.5) (adj.) Ref. group: ≥2500 g	Hwang, 2013 (36)	411/30,025
	Birth weight *z* score	*z* score <−2: ASD+/ID−: OR 9.9 (3.3, 30)^∗^ ASD+/ID^+^: OR 2.1 (0.5, 9.9) ASD−/ID+: OR 2.0 (0.7, 5.3) *z* score ≥−2, <1: NS *z* score ≥1: NS	Joseph, 2017 (23)	59^§^/840
	Birth weight (≤750, 751–1000, >1000 g)	NS	Joseph, 2017 (23)	59^§^/840
	Birth weight, grams	RR 0.75 (0.51, 1.09)	Bakian, 2018 (31)	112/4855
	Head circumference *z* score (<−2, ≥−2 < 1, ≥1)	NS	Joseph, 2017 (23)	59^§^/840
	Large for gestational age	90–95th percentile: 23–31 weeks + 6 days: OR 0.49 (0.25, 0.95)^∗^ 32–33 weeks + 6 days: OR 0.56 (0.25, 1.26) 34–36 weeks + 6 days: OR 1.04 (0.84, 1.30) >95th percentile: 23–31 weeks + 6 days: OR 0.45 (0.21, 0.95)^∗^ 32–33 weeks + 6 days: OR 1.00 (0.52, 1.89) 34–36 weeks + 6 days: OR 0.97 (0.77, 1.22) Ref. group: appropriate for GA, >10th percentile–<90th percentile	Moore, 2012 (34)	2273/515,789
	Low birth weight	OR 1.664 (1.249, 2.216)^∗^	Ha, 2014 (41)	372/9302
	Small for gestational age	<5th percentile: 23–31 weeks + 6 days: OR 1.60 (1.09, 2.35)^∗^ 32–33 weeks + 6 days: OR 1.83 (1.16, 2.87)^∗^ 34–36 weeks + 6 days: OR 1.07 (0.86, 1.34) 5th–10th percentile: 23–31 weeks + 6 days: OR 1.36 (0.91, 2.02) 32–33 weeks + 6 days: OR 1.00 (0.57, 1.78) 34–36 weeks + 6 days: OR 1.12 (0.91, 1.38) Ref. group: appropriate for GA, >10th percentile–<90th percentile	Moore, 2012 (34)	2273/515,789
Maternal Health Factors				
Fever (Maternal)	Fever during delivery	ASD+/ID−: OR 3.6 (0.98, 13) ASD+/ID+: OR 0.6 (0.1, 4.4) ASD−/ID+: OR 2.9 (1.2, 6.7)^∗^	Joseph, 2017 (23)	59^§^/840
	Fever during pregnancy	NS	Joseph, 2017 (23)	59^§^/840
Infection (Maternal)	Maternal histological chorioamnionitis	Stage 1 (early): OR 0.51 (0.19, 1.34) Stage 2 (mod.): OR 1.08 (0.72, 1.62) Stage 3 (adv.): OR 1.65 (0.92, 2.94) Grade 1 (mild/mod.): OR 0.71 (0.31, 1.64) Grade 2 (severe): OR 1.30 (0.69, 2.48)	Venkatesh, 2020 (30)	56/773
	Periodontal infection	NS	Joseph, 2017 (23)	59^§^/840
	Urinary tract infection	NS	Joseph, 2017 (23)	59^§^/840
	Vaginal/cervical infection	ASD+/ID−: OR 0.9 (0.2, 4.1) ASD+/ID+: OR 2.7 (1.2, 6.4)^∗^ ASD−/ID+: OR 0.7 (0.3, 1.6)	Joseph, 2017 (23)	59^§^/840
Preeclampsia	Preeclampsia	NS	Getahun, 2017 (32)	524/27,949
	Preeclampsia	NS	Joseph, 2017 (23)	59^§^/840
Membrane Rupture	Duration of membrane rupture (<1, 1–2, >24 hours) (latency between membrane rupture and delivery)	NS	Joseph, 2017 (23)	59^§^/840
	Preterm prelabor membrane rupture	NS	Joseph, 2017 (23)	59^§^/840
Placental Abruption	Placental abruption	NS	Getahun, 2017 (32)	524/27,949
	Placental abruption	NS	Joseph, 2017 (23)	59^§^/840
Cesarean Section	Cesarean	NS	Joseph, 2017 (23)	59^§^/840
	Cesarean section (compared to vaginal delivery)	GA weeks 26–36: OR 1.42 (1.23, 1.64)^∗^	Yip, 2017 (38)	2160/24,042
	Emergency cesarean section (compared to vaginal delivery)	GA weeks 26–30: OR 0.86 (0.58, 1.26) GA week 31: OR 1.46 (0.74, 2.87) GA week 32: OR 1.24 (0.73, 2.12) GA week 33: OR 1.18 (0.74, 1.90) GA week 34: OR 1.23 (0.86, 1.76) GA week 35: OR 1.00 (0.72, 1.38) GA week 36: OR 1.31 (1.04, 1.63)^∗^	Yip, 2017 (38)	2160/240,042
	Planned cesarean section (compared to vaginal delivery)	GA weeks 26–30: OR 1.14 (0.81, 1.60) GA week 31: OR 1.38 (0.74, 2.58) GA week 32: OR 1.25 (0.76, 2.05) GA week 33: OR 1.10 (0.69, 1.74) GA week 34: OR 0.86 (0.58, 1.29) GA week 35: OR 1.18 (0.86, 1.61) GA week 36: OR 1.32 (1.05, 1.65)^∗^	Yip, 2017 (38)	2160/240,042
Smoking	Passive smoke exposure	NS	Joseph, 2017 (23)	59^§^/840
	Smoked during pregnancy	NS	Joseph, 2017 (23)	59^§^/840
Maternal Magnesium	Antenatal magnesium administration	RR 0.79 (0.47, 1.34)	Bakian, 2018 (31)	112/4855
	Magnesium sulfate (for tocolysis; seizure prophylaxis)	NS	Joseph, 2017 (23)	59^§^/840
Maternal Medications	Acetaminophen (maternal)	NS	Joseph, 2017 (23)	59^§^/840
	Antenatal steroid course (complete, partial, none)	NS	Joseph, 2017 (23)	59^§^/840
	Antibiotic (maternal)	ASD+/ID−: OR 0.1 (0.01, 0.7)^∗^ ASD+/ID+: OR 0.8 (0.4, 1.9) ASD+/ID+: OR 1.3 (0.9, 2.3)	Joseph, 2017 (23)	59^§^/840
	Aspirin (maternal)	NS	Joseph, 2017 (23)	59^§^/840
	NSAID (maternal)	NS	Joseph, 2017 (23)	59^§^/840
Maternal Health (Miscellaneous)	Diabetes (diabetes mellitus and gestational diabetes)	RR 1.24 (0.68, 2.25)	Bakian, 2018 (31)	112/4855
	Highest white blood cell count (maternal)	NS	Joseph, 2017 (23)	59^§^/840
	Prepregnancy BMI (<18.5, 18.5 − <25, 25 − <30, ≥30)	NS	Joseph, 2017 (23)	59^§^/840
Pregnancy-/Delivery-Related Outcomes (Miscellaneous)	Intrapartum condition^b^	NS	Getahun, 2017 (32)	524/27,949
	>1 intrapartum condition^b^	NS	Getahun, 2017 (32)	524/27,949
	Antepartum condition^b^	NS	Getahun, 2017 (32)	524/27,949
	Antepartum or intrapartum condition^b^	NS	Getahun, 2017 (32)	524/27,949
	Antepartum and intrapartum condition^b^	Increased risk^∗^	Getahun, 2017 (32)	524/27,949
	Cervical insufficiency	NS	Joseph, 2017 (23)	59^§^/840
	Duration of labor (0, ≤12, >12 hours)	NS	Joseph, 2017 (23)	59^§^/840
	Fetal dystocia	NS	Getahun, 2017 (32)	524/27,949
	Fetal indication	NS	Joseph, 2017 (23)	59^§^/840
	First pregnancy	NS	Joseph, 2017 (23)	59^§^/840
	Malpresentation	NS	Getahun, 2017 (32)	524/27,949
	Preterm labor	NS	Joseph, 2017 (23)	59^§^/840
	Prolapsed/nuchal cord	NS	Getahun, 2017 (32)	524/27,949
	Singleton	NS	Joseph, 2017 (23)	59^§^/840
	Vaginal breech delivery (compared to vaginal cephalic delivery)	GA 24–27 weeks + 6 days: OR 0.50 (0.11, 2.31) GA 28–31 weeks + 6 days: OR 1.51 (0.49, 4.63) GA 32–36 weeks + 6 days: OR 2.28 (1.14, 4.56)^∗^	Toijonen, 2021 (37)	116/23,803
	Years since last pregnancy (<1, 1–2, 2+)	NS	Joseph, 2017 (23)	59^§^/840
Maternal, Other (Miscellaneous)	Maternal age (<21, 21–35, >35)	NS	Joseph, 2017 (23)	59^§^/840
	Medicaid insurance	RR 0.94 (0.59, 1.50)	Bakian, 2018 (31)	112/4855
	Public insurance	NS	Joseph, 2017 (23)	59^§^/840
	Relationship status (single, not single)	NS	Joseph, 2017 (23)	59^§^/840
	Self-supported	NS	Joseph, 2017 (23)	59^§^/840
	Years of education (≤12, >12 − <16, ≥16)	NS	Joseph, 2017 (23)	59^§^/840
Child Outcome				
Brain Hemorrhage	Intracranial hemorrhage	Grade 1/2: HR 1.9 (1.1, 3.4)^∗^ Grade 3/4: HR 3.4 (1.4, 8.6)^∗^ Ref. group: none or ultrasound not done	Kuzniewicz, 2014 (33)	80/3807
	Intraventricular hemorrhage	OR 1.0 (0.5, 1.8)	Hwang, 2013 (36)	411/30,025
	Isolated intraventricular hemorrhage	OR 1.39 (0.69, 2.78) (crude), 1.24 (0.59, 2.6) (adj.)	Campbell, 2021 (29)	29/858
	Intraventricular hemorrhage and white-matter damage	OR 0.97 (0.34, 2.79) (crude), 0.58 (0.19, 1.77) (adj.)	Campbell, 2021 (29)	29/858
Respiratory Problems	Birth asphyxia	NS	Getahun, 2017 (32)	524/27,949
	Birth asphyxia	OR 1.2 (0.8, 1.8)	Hwang, 2013 (36)	411/30,025
	Bronchopulmonary dysplasia	OR 2.2 (1.2, 4.1) (crude)^∗^, 1.5 (0.8, 2.9) (adj.)	Hwang, 2013 (36)	411/30,025
	High frequency ventilation	HR 2.2 (1.1, 4.6)	Kuzniewicz, 2014 (33)	80/3807
	Mechanical ventilation	HR 1.2 (0.7, 2.0)	Kuzniewicz, 2014 (33)	80/3807
	Respiratory distress	OR 0.6 (0.4, 1.1) (a), 0.7 (0.4, 1.2) (b)	Buchmayer, 2009 (40)	119/502
	Respiratory distress syndrome	OR 1.2 (1.0, 1.5)	Hwang, 2013 (36)	411/30,025
Jaundice (Neonatal)	Hyperbilirubinemia	OR 1.0 (0.8, 1.2)	Hwang, 2013 (36)	411/30,025
	Neonatal hyperbilirubinemia	HR 0.71 (0.36, 1.39) (crude), 0.72 (0.37, 1.42) (adj.)	Hung, 2021 (35)	48/4468
	Neonatal jaundice	OR 0.7 (0.4, 1.1) (adj. a) 0.7 (0.5, 1.2) (adj. b)	Buchmayer, 2009 (40)	119/502
	Neonatal jaundice	OR 1.22 (0.94, 1.57) (crude), 1.84 (1.06, 3.20) (adj.)^∗^	Cordero, 2020 (39)	119/410
Inflammatory Markers (Neonatal)	Regulated upon activation normal t-cell expressed and secreted (RANTES; CCL5)	NS	Korzeniewski, 2018 (24)	36/763
	Angiopoietin-1 (ANG-1)	NS	Korzeniewski, 2018 (24)	36/763
	Angiopoietin-2 (ANG-2)	NS	Korzeniewski, 2018 (24)	36/763
	Brain-derived neurotrophic factor (BDNF)	NS	Korzeniewski, 2018 (24)	36/763
	C-reactive protein	NS	Korzeniewski, 2018 (24)	36/763
	Erythropoietin (EPO)	NS	Korzeniewski, 2018 (24)	36/763
	IL-6	Day 1: NS. Day 7: NS. Day 14: NS. Day 21: NS. Day 28: NS. Two of postnatal days 1, 7, 14: NS. Both postnatal days 21, 28: OR 2.6 (1.03, 6.4)^∗^	Korzeniewski, 2018 (24)	36/763
	IL-6 receptor (IL-6R)	NS	Korzeniewski, 2018 (24)	36/763
	IL-8 (CXCL8)	NS	Korzeniewski, 2018 (24)	36/763
	Insulin-like growth factor binding protein-1 (IGFBP-1)	NS	Korzeniewski, 2018 (24)	36/763
	Intercellular adhesion molecule-1 (ICAM-1; CD54)	NS	Korzeniewski, 2018 (24)	36/763
	Interleukin 1β (IL-1β)	NS	Korzeniewski, 2018 (24)	36/763
	Metalloproteinase (MMP)-9	Day 1: NS. Day 7: NS. Day 14: OR 0.2 (0.05, 0.8)^∗^ Day 21: NS. Day 28: NS. Two of postnatal days 1, 7, 14: NS. Both postnatal days 21, 28: NS.	Korzeniewski, 2018 (24)	36/763
	Myeloperoxidase	NS	Korzeniewski, 2018 (24)	36/763
	neurotrophin-4 (NT-4)	Day 1: NS. Day 7: NS. Day 14: NS. Day 21: OR 2.1 (1.01, 4.3)^∗^ day 28: NS. Two of postnatal days 1, 7, 14: NS. Both postnatal days 21, 28: increased risk (OR missing)^∗^	Korzeniewski, 2018 (24)	36/763
	Placenta growth factor (PIGF)	NS	Korzeniewski, 2018 (24)	36/763
	Serum amyloid A (SAA)	Day 1: NS. Day 7: NS. Day 14: OR 2.5 (1.1, 5.5)^∗^ Day 21: NS. Day 28: NS. Two of postnatal days 1, 7, 14: OR 2.5 (1.2, 5.3)^∗^ Both postnatal days 21, 28: NS.	Korzeniewski, 2018 (24)	36/763
	Thyroid-stimulating hormone	NS	Korzeniewski, 2018 (24)	36/763
	TNF receptor-1 (TNFR-1)	NS	Korzeniewski, 2018 (24)	36/763
	TNF receptor-2 (TNFR-2)	NS	Korzeniewski, 2018 (24)	36/763
	Tumor necrosis factor-α (TNF-α)	Day 1: NS. Day 7: NS. Day 14: NS. Day 21: NS. Day 28: OR 2.5 (1.2, 5.2)^∗^ Two of postnatal days 1, 7, 14: NS. Both postnatal days 21, 28: NS.	Korzeniewski, 2018 (24)	36/763
	Vascular cell adhesion molecule-1 (VCAM-1; CD106)	NS	Korzeniewski, 2018 (24)	36/763
	Vascular endothelial growth factor (VEGF)	NS	Korzeniewski, 2018 (24)	36/763
	VEGF receptor-1 (VEGFR-1 also known as SFLT-1)	NS	Korzeniewski, 2018 (24)	36/763
	VEGF receptor-1 (VEGFR-2 [KDR])	NS	Korzeniewski, 2018 (24)	36/763
	Basic fibroblastic growth factor (BFGF)	NS	Korzeniewski, 2018 (24)	36/763
	IL-10	Day 21: OR 1.2 (0.5, 3.1) Day 28: OR 2.1 (0.9, 5.0) Both days: OR 1.8 (0.7, 5.0)	Leviton, 2018 (26)	XX/449
	IL-4	Day 21: OR 1.5 (0.6, 3.6) Day 28: OR 2.4 (1.04, 5.7)^∗^ Both days: OR 1.2 (0.4, 3.5)	Leviton, 2018 (26)	XX/449
Neonatal Health (Miscellaneous)	Bacteremia	HR 1.6 (0.8, 3.4)	Kuzniewicz, 2014 (33)	80/3807
	Cerebral dysfunction^a^	OR 4.03 (1.5, 11.1) (crude)^∗^, 4.7 (1.7–13.0) (adj.)^∗^	Hwang, 2013 (36)	411/30,025
	Cystic periventricular leucomalacia	HR 1.7 (0.2, 12.4)	Kuzniewicz, 2014 (33)	80/3807
	Fetal histological chorioamnionitis	Stage 1 (early): OR 1.85 (0.48, 7.17) Stage 2 (mod.): OR 1.82 (0.75, 4.43) Stage 3 (adv.): OR 1.58 (0.63, 4.05) Grade 1 (mild/mod.): OR 1.69 (1.01, 2.88)^∗^ Grade 2 (severe): NA	Venkatesh, 2020 (30)	56/773
	Hypoglycemia	OR 1.3 (0.5, 3.3) (a), 1.3 (0.5, 3.3) (b)	Buchmayer, 2009 (40)	119/502
	Hypothyroidism	OR 2.2 (0.5, 8.8)	Hwang, 2013 (36)	411/30,025
	Isolated white-matter damage	OR 1.02 (0.23, 4.42) (crude), 0.74 (0.09, 5.88) (adj.)	Campbell, 2021 (29)	29/858
	Necrotizing enterocolitis	NA	Kuzniewicz, 2014 (33)	80/3807
	Patent ductus arteriosus	OR 1.2 (0.9, 1.7)	Hwang, 2013 (36)	411/30,025
	Transfusion	HR 1.4 (0.7, 2.7)	Kuzniewicz, 2014 (33)	80/3807
Score for Neonatal Acute Physiology (SNAP-II)	SNAP-II score ≥30	OR ∼ 2.6 (1.3, 5.0) (crude)^∗^, ∼2.0 (0.95, 4.2) (adj.)	Logan, 2017 (27)	59/874
	SNAP-II score 20–29	OR ∼ 2.5 (1.3, 4.85) (crude)^∗^, ∼2.3 (1.1, 4.6) (adj.)^∗^	Logan, 2017 (27)	59/874
Resuscitation (Neonatal)	Delivery room epinephrine or chest compressions	HR 1.1 (0.4, 3.1)	Kuzniewicz, 2014 (33)	80/3807
	Neonatal resuscitation	NS	Getahun, 2017 (32)	524/27,949
Magnesium	Fetal serum magnesium, mg/dL	RR 1.15 (0.86, 1.53)	Bakian, 2018 (31)	112/4855
Neonatal Medications	Acid suppressant use ≤24 months of age	OR 2.18 (1.37, 3.49) (crude), 1.84 (1.15, 2.95) (adj.)^∗^	Jensen, 2022 (28)	61/889
	Inotropic support (neonatal)	HR 1.4 (0.7, 2.8)	Kuzniewicz, 2014 (33)	80/3807

Adj. indicates adjusted for covariates, and crude indicates not adjusted for covariates. NS indicates not statistically significant and manuscript did not display effect estimate.

ASD, autism spectrum disorder; GA, gestational age at birth; HR, hazard ratio; ID, intellectual disability; LBW, low birth weight; OR, odds ratio; RR, relative risk; XX, missing information.

∗Statistically significant.

§ASD+/ID-: *n* = 27; ASD+/ID+: *n* = 32; ASD-/ID+: *n* = 71.

a ICD-9-CM codes for significant cerebral dysfunction in newborns: 779.0 (Convulsions in newborn), 779.1 (Other and unspecified cerebral irritability in newborn), 779.2 (Cerebral depression, coma, and other abnormal cerebral signs in fetus or newborn).

b Intrapartum conditions: breech/transverse fetus, fetal dystocia, prolapsed/nuchal cord, birth asphyxia; antepartum conditions: placental abruption, preeclampsia.

### Gestational Age and Preterm Birth

The 8 ELGAN studies investigated infants who were born extremely preterm (<28 weeks), determined using dates of embryo retrieval, intrauterine insemination, fetal ultrasound, information on last menstrual period, or neonatal intensive care unit logs. Four other studies determined GA based on ultrasounds and/or information on last menstrual period, with GA ranges varying between <37 weeks (40), 26 to 36 weeks (38), 24 to 36 weeks (37), and 24 to 34 weeks (33). Two studies relied on ICD-9 codes related to birth at <37 weeks GA (35,36). One study relied on parental recall of children being born at least 3 weeks before their reported due date (41). Four studies did not elucidate ascertainment strategy for GA ranges 35 to 37 weeks (39), 28 to 36 weeks (32), 23 to 37 weeks (34), and 22 to 36 weeks (31) (Table S3). Gestational weeks were categorized according to clinical classifications where applicable: extremely preterm (<28 weeks), very preterm (28–32 weeks), moderately preterm (32–34 weeks), and late preterm (34–37 weeks).

### Risk Factors for ASD

The following section describes risk factors for ASD in preterm samples that were statistically significant in at least 1 study. All risk factors that were examined in the literature are detailed in Table 1.

Fetal respiratory problems were linked to increased risk of ASD in 2 studies (33,36). Bronchopulmonary dysplasia, or chronic lung disease, was associated with increased risk of ASD before adjustment for confounding factors, but not after (36). Also, use of a ventilator, which many infants who eventually develop bronchopulmonary dysplasia are treated with, was linked to ASD risk in another study (33); however, this was only true for high-frequency ventilation (used for severe cases) and not for mechanical ventilation more generally. Other respiratory problems, including birth asphyxia (32,36), respiratory distress (40), and respiratory distress syndrome (36) were not associated with ASD risk.

Brain hemorrhage, particularly intracranial hemorrhage, was associated with increased risk of ASD at the milder bleeding grades 1 and 2 (hazard ratio [HR] 1.9; 95% CI, 1.1–3.4) and potentially even higher risk at the more serious grades 3 and 4 (HR 3.4; 95% CI, 1.4–8.6) in a retrospective cohort study of 11 U.S. hospitals (33). Intraventricular hemorrhage was not associated with ASD in 2 studies (29,36). Additional information on cerebellar hemorrhages was not available in the included studies.

Significant cerebral dysfunction, which was defined as convulsions, other and unspecified cerebral irritability, cerebral depression, coma, and other abnormal cerebral signs, was associated with increased risk of ASD with and without adjustment in a nationwide study in Taiwan (36).

In 1 study (39), neonatal jaundice was associated with increased ASD risk after adjustment but not before. Jaundice was not linked to ASD risk in 3 other studies with and without adjustment (35,36,40).

One study (27) investigated Score for Neonatal Acute Physiology (SNAP-II), which is a physiology-based indicator of endogenous mortality risk from the first 12 postnatal hours (scores range 0–115, from low to high risk). SNAP-II scores of 20 to 29 were associated with increased risk of ASD before and after adjustment. Scores of ≥30 were linked to increased ASD risk only before adjustment.

In an ELGAN study (individuals hereafter referred to as ELGANs), mild to moderate grade fetal histological chorioamnionitis (fetal systemic inflammatory response based on umbilical cord inflammation [funisitis]) was linked to increased risk of ASD; however, it was not associated with ASD by stage (early, moderate, or advanced) (30). In the same study, maternal histological chorioamnionitis (histologically acute inflammation at the chorionic plate) was not associated with child’s ASD at any stage or grade.

Several inflammatory markers in ELGANs were linked to increased risk of ASD. These include elevated levels of interleukin 6 (IL-6) on postnatal days (PNDs) 21 and 28; neurotrophin-4 (NT-4) on PND 21 and 28; serum amyloid A on two of PNDs 1, 7, or 14; and tumor necrosis factor-α (TNF-α) on PND 28 (24), as well as top quartile levels of interleukin 4 (IL-4) on PND 14 (26). Metalloproteinase-9 on PND 14 was linked to decreased risk of ASD (24). (See Table 1 for the full list of inflammatory markers, including all those not associated with ASD.)

Maternal health complications yielded mixed results. Most outcomes came from a single study of ELGANs, in which maternal vaginal/cervical infection was linked to increased risk of ASD with co-occurring intellectual disability (ID), but not ASD without co-occurring ID (23). In the same study, maternal periodontal infection and urinary tract infection were not associated with ASD, nor were maternal fever during pregnancy or delivery, highest white blood cell count, prepregnancy body mass index, or preeclampsia. Preeclampsia was also not associated with ASD in another study (32), and neither was diabetes (preexisting or gestational) (31).

Several studies investigated medication use. In ELGANs, maternal use of acetaminophen, antenatal corticosteroids, aspirin, and nonsteroidal anti-inflammatory medication during pregnancy was not linked to ASD risk; meanwhile, maternal antibiotic use (unadjusted) was linked to decreased risk of ASD without co-occurring ID but was not associated with ASD with co-occurring ID (23). Acid suppressant use at ≤24 months was linked to increased risk of ASD (28). In a non-ELGAN cohort, neonatal inotropic support (dopamine, dobutamine, or epinephrine infusions) was not associated with ASD risk (33).

Compared to vaginal cephalic delivery, vaginal breech delivery was associated with increased risk of ASD in late-preterm-born children (born GA 32–36 weeks) (37). This was not found for the earlier GA groups (24–27 or 28–31 weeks).

Cesarean section was linked to increased risk of ASD compared to vaginal delivery in a large register-based study of 4 Nordic countries and Western Australia (38), but not in the ELGAN cohort (unadjusted) (23). In the former study, when stratified by emergency versus planned cesarean section, both were associated with ASD risk among individuals who were born at 36 weeks GA but not earlier.

Male sex was linked to increased risk of ASD in preterm (31,36) and extremely preterm (25) samples. In an ELGAN study (23), male sex was linked to increased risk of ASD with co-occurring ID but not ASD without co-occurring ID.

The relationship between low birth weight (LBW) and ASD in preterm infants remains unclear, largely due to inconsistent birth weight classifications used across studies. Nevertheless, several studies have linked LBW to a higher risk of ASD (36,41), while two did not (23,31). Additionally, being small for gestational age (SGA, which is a function of both birth weight and GA) was associated with increased risk of ASD, whereas being large for gestational age was associated with decreased risk (34). Having a birth weight *z* score of <−2 was significantly associated with ASD without co-occurring ID but not with ASD with co-occurring ID (23).

### Meta-analyses

**Figure 3:**
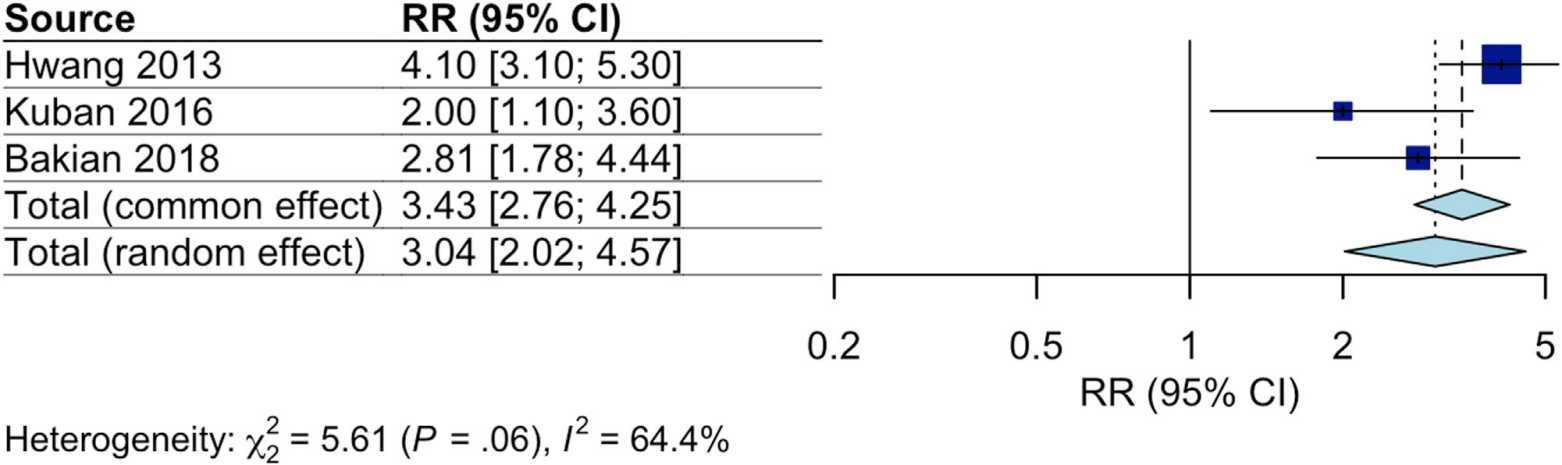
Meta-analysis of the association between autism spectrum disorder and male sex in individuals born preterm. RR, relative risk.

Four (23,25,31,36) studies examined the association between male sex and ASD using consistent exposure definitions and effect measures. Two (23,25) used the same sample, and thus one (23) was removed. When combined in a meta-analysis, male sex was positively associated with ASD in individuals who were born preterm (RR 3.04, 95% CI, 2.02–4.57) (Figure 3). Heterogeneity between studies was substantial (*I*^*2*^ = 64%).

## Discussion

Using a comprehensive, preregistered search strategy, we synthesized 19 population-based studies that examined risk factors for ASD in individuals who were born preterm. The literature suggests that male sex is a strong risk factor for ASD in preterm-born individuals. Findings on LBW were inconclusive, and neonatal jaundice is not associated with ASD. Sex was the only risk factor examined using consistent methodology in more than 2 studies, qualifying it for meta-analysis. LBW and jaundice were examined in various studies, but discordant measures were used. The remaining wide variety of examined exposures were not replicated. Systematic and comprehensive investigations of risk factors for ASD specifically among individuals who were born preterm are lacking, which impedes our ability to examine the determinants of ASD in this high-risk group.

**Figure 4:**
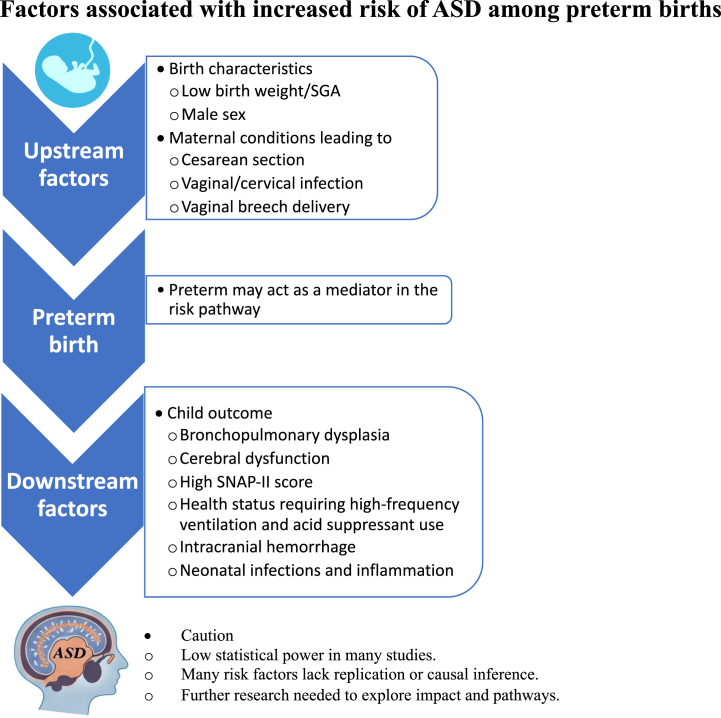
Overview of the key take-home message. ASD, autism spectrum disorder; SGA, small for gestational age; SNAP, Score for Neonatal Acute Physiology.

GA at birth is influenced by various upstream factors that could act upon both the risk of preterm birth and ASD. These include male sex, LBW/SGA, as well as maternal conditions that cause cesarean section, vaginal/cervical infection, and vaginal breech delivery, which were associated with a higher risk of ASD in the current study. The downstream consequences of premature birth may also mediate the association between preterm birth and ASD, including intracranial hemorrhage, bronchopulmonary dysplasia, cerebral dysfunction, high SNAP-II score, health status requiring high-frequency ventilation, acid suppressant use, and neonatal infections and inflammation (IL-6, NT-4, serum amyloid A, TNF-α, IL-4, and fetal histological chorioamnionitis). In the 5 studies that provided information on infants born preterm and at term (32,35,38, 39, 40), certain risk factors increased ASD risk only among those who were born at term, suggesting that preterm birth itself could play a role in the causal pathway. However, statistical power was generally low, and thus we caution against drawing conclusions from these comparisons. Considering that many of these risk factors were studied without replication or attempt at causal inference, the impact and acting pathways of these findings are still unknown and should be explored in larger studies (see Figure 4 for a summary of the key take-home message).

The association between male sex and ASD risk is robust in both preterm (23,25,31,36) and general (42,43) samples. Our meta-analysis indicates a >3-fold risk of ASD in male children who are born preterm, which is similar to non-exclusively-preterm estimates [i.e., a 3–4-fold risk (43)]. Notably, population estimates in exclusively term-born children are lacking. Despite our findings that were consistent with general estimates, alternative evidence suggests that the association between GA and ASD may differ meaningfully by sex. In an Israeli study (44), the risk of ASD was higher in females than in males at extreme prematurity, which then decreased with increasing GA up to 37 weeks; meanwhile, risk was stably elevated across GA in males. In a much larger, Nordic register-based study (45), the RR of ASD in offspring born moderate-to-late preterm was significantly higher in females than in males born in those weeks. Although not statistically significant, the risk point estimate was also higher in females who were born extremely and very preterm. Other identified sex differences include higher rates of perinatal mortality, morbidity, and poorer outcomes in male infants born preterm (46).

In general, the evidence suggests an association between LBW and ASD. Notably (and rather concerningly), despite it being such a critical risk factor, the inconsistent definitions of LBW across studies has resulted in mixed and inconclusive inferences about the nature of the relationship. For example, one study found that increased ASD risk was associated with birth weights <750 g and 750 to 1499 g, but not to birth weights 1500 to 2499 g (36). Another study without adjustment reported an association between ASD and birth weight <2500 g (41). In an ELGAN study, categorizing birth weight as ≤750 g, 751 to 1000 g, and >1000 g yielded no association with ASD (23). Another study corroborated this null finding for birth weight (31). Similarly, the impact of SGA (<5th percentile) on ASD risk varied by GA; SGA was associated with increased risk in infants born at extremely, very, and moderate preterm, but not late preterm (34). SGA in the 5th to 10th percentile did not show an association with ASD. Conversely, large for gestational age (>95th percentile) was associated with decreased ASD risk in extreme and very preterm births but not in moderate-to-late preterm births. In an ELGAN study (23), having a birth weight *z* score of <−2 was associated with ASD without co-occurring ID, but with a very wide confidence interval. No association was found between birth weight *z* score and ASD with co-occurring ID. Despite critiques of LBW and SGA as weak proxies for fetal growth restriction (47,48), the association between LBW and ASD may reflect immaturity of brain development in utero, where the pathophysiology that limits fetal growth also affects neurological development. For example, consequences of placental insufficiency include primary fetal growth restriction (49), acidosis (49), and hypoxic ischemic encephalopathy (50), each of which contributes to risk of ASD (51). Nevertheless, LBW should be considered with caution because being born below a certain cutoff weight is not necessarily indicative of poor fetal health, and inversely, being born above this cutoff does not rule out the possibility that fetal growth did or will not reach its full potential.

### Strengths and Limitations

A strength of this review is its comprehensive search strategy developed professionally by a librarian, encompassing literature from 3 major databases. Its use of exclusively population-based research makes this a unique synthesis of information with an emphasis on the representativeness of the included samples. Furthermore, this work is a collaboration between a large interdisciplinary team of epidemiologists and clinical experts.

Despite the considerable sample sizes of many of the included studies, the limitations of the current review still include limited statistical power [∼10% of babies are born preterm (52), 6%–20% of whom develop ASD (6)]. Findings were rarely replicated across studies, and when they were, measures were often mismatched (e.g., continuous vs. categorical measures) or were measured by different proxies, thereby limiting comparability. Furthermore, covariate adjustment varied substantially between studies, adjustment for perinatal complications was rare, and familial confounding of ASD was rarely considered.

An important limitation involves having conditioned on preterm birth and potentially introducing collider bias (53), wherein preterm birth and ASD are influenced by the same variables (e.g., certain maternal infections), thus generating inflated associations between those variables and ASD. Additionally, the varying diagnostic criteria for ASD between studies raises the potential issue of misclassification bias. Survival bias may affect the accuracy of estimates because many babies who are born prematurely are at increased risk of death, birth defects, and other disorders/diseases, which can lead to underestimation of ASD risk when children are censored due to these other serious conditions preceding ASD. Surveillance bias poses an issue in such research, especially for individuals who are born very early, who are subject to intensive follow-up procedures that increase the likelihood and speed of detecting milder forms of ASD. Furthermore, confounding by maternal age (related to both preterm birth and ASD) and calendar time (reflecting secular trends in GA management, maternal age, and ASD diagnoses) was often unaddressed. Additionally, publication bias pervades research regardless of topic because neutral studies are far less likely to be published.

Importantly, the literature does not distinguish between spontaneous and induced preterm birth. Spontaneous preterm delivery, including preterm premature rupture of membranes, is associated mainly with inflammation/infections (54) and with uterine overdistension (55), elevated neuro-endocrine activity/homeostasis disruptions (56), and genetic causes (57). Reasons for induced preterm birth include intrauterine growth restriction and maternal conditions such as preeclampsia (54). Therefore, the risk factors that underlie or are initiated by spontaneous versus induced preterm birth may represent different pathways of risk of ASD. Finally, confounding effects may be present in the association between male sex and ASD due to a higher incidence of preterm delivery in pregnancies carrying male fetuses (58).

### Future Research

Individuals who are born preterm have an increased risk of ASD compared to individuals who are born at term and may represent a different etiologic group, environmentally and genetically, in terms of neurodevelopment. Considering that the elevated risk of ASD in infants who are born preterm may be explained in part by the presence of cognitive impairment linked to premature birth, it is critical that future studies specifically examine the prevalence of co-occurring conditions and phenotypes, including ID, cognitive impairment, and nonverbal outcomes. While this review focused exclusively on the preterm population, future work should aim to compare risk factors for ASD between individuals who are born preterm and full term, aiming to distinguish between risk that derives from preterm birth itself and the risk factors associated with preterm birth. Therefore, leveraging large, population-based studies with robust designs is imperative to navigate the complex methodological issues concerning the identification of causal pathways between preterm birth and ASD.

### Conclusions

There are major gaps in knowledge about risk for ASD in individuals who are born preterm. Male sex is the only persistent risk factor for ASD across studies, and although some evidence exists for additional factors, they remain understudied. Continued research is needed to better understand the determinants of ASD in individuals who are born preterm.

## Supplementary Material

Supplemental Methods and Materials and Tables S1–S3

Key Resources Table
