# Supplementary material for: Risk Factors for Autism Spectrum Disorder in Individuals Born Preterm: A Systematic Review and Meta-Analysis of Population-Based Studies
Source: Biol Psychiatry Glob Open Sci. 2025 May 14;5(5):100535. doi: 10.1016/j.bpsgos.2025.100535 (PMC12268552; doi:10.1016/j.bpsgos.2025.100535)
Supplement: Supplemental Methods and Materials and Tables S1–S3 [file mmc1.pdf]

SUPPLEMENTARY INFORMATION

Risk Factors for Autism Spectrum Disorder in Individuals Born Preterm: A Systematic Review and Meta-Analysis of Population-Based Studies

Yang *et al.*

Contents:

Supplementary Methods 2

Supplementary Table 1: Database search strategies and inclusion + exclusion criteria 3

Supplementary Table 2: Newcastle-Ottawa Scale to assess the quality of included studies 4

Supplementary Table 3: Characteristics of the studies included in the systematic review and meta-analysis. 6

Supplementary Methods

Eligibility criteria

To maximize the generalizability of our findings, only population-based studies were included, without geographic restrictions. Original, English-language research examining risk factors for ASD in preterm cohorts were included. Studies with unreliable ascertainment for ASD (e.g., use of a single screening tool) were excluded.

Systematic literature search

The search strategy was developed by a librarian from the Karolinska Institutet University Library (GBK) (**Supplementary Table 1**).

The search was initially conducted in Ovid MEDLINE, Embase, and Web of Science on September 23, 2020. Title and abstract screening were performed simultaneously by four study authors (MSMP, ND, NZ, and TF) using Rayyan software (1). Full-text screening was conducted first independently and then in pairs of reviewers. Disagreements were resolved through discussion. To compensate for delays caused by the COVID-19 pandemic, the search was updated to include literature published up to September 25, 2023, using the same search strategy. Article screening was done in the same method as before by two study authors (BY and NZ).

Data extraction and study quality assessment

Data extraction and study quality assessment were conducted independently and then jointly by two researchers. Study quality was assessed using the validated Newcastle-Ottawa scale (2). Low quality studies were excluded from meta-analyses.

Supplementary Table 1: Database search strategies and inclusion + exclusion criteria

Database and search strategy:
**Ovid MEDLINE(R) and Epub Ahead of Print, In-Process & Other Non-Indexed Citations**<br>**01 Jan. 1946 - 25 Sept. 2023**<br>1. exp Autism Spectrum Disorder/<br>2. (Asperger* or autis* or ASD).ti,ab,kf.<br>3. or/1-2<br>4. Premature Birth/<br>5. exp Infant, Premature/<br>6. exp Infant, Low Birth Weight/<br>7. Gestational Age/<br>8. ((premature or preterm or pre-term) adj3 (birth or child* or delivery or infant* or neonat*)).ti,ab,kf.<br>9. (infant* adj3 (birth weight or gestation* age)).ti,ab,kf.<br>10. ((fetal or gestation*) adj3 (age or growth or maturity)).ti,ab,kf.<br>11. or/4-10<br>12. 3 and 11<br>**Embase**<br>**01 Jan. 1910 - 25 Sept. 2023**<br>('autism'/exp/mj OR asperger*:ti,ab,kw OR autis*:ti,ab,kw OR asd:ti,ab,kw)<br>AND<br>('prematurity'/mj OR 'low birth weight'/exp/mj OR 'gestational age'/mj OR (((premature OR preterm OR 'pre term') NEAR/3 (birth OR child* OR delivery OR infant* OR neonat*)):ti,ab,kw) OR ((infant* NEAR/3 ('birth weight' OR 'gestation* age')):ti,ab,kw) OR (((fetal OR gestation*) NEAR/3 (age OR growth OR maturity)):ti,ab,kw))<br>**Web of Science**<br>**01 Jan. 1956 - 25 Sept. 2023**<br>#1: TOPIC: ((Asperger* or autis* or ASD))<br>#2: TOPIC: (((premature or preterm or pre-term) NEAR/3 (birth or child* or delivery or infant* or neonat*))) OR TOPIC: ((infant* NEAR/3 ("birth weight" or "gestation* age"))) OR TOPIC: (((fetal or gestation*) NEAR/3 (age or growth or maturity)))<br>#3: #1 AND #2
**Inclusion criteria:**<br>1. Cohort, case-control, and cross-sectional studies that examined the associations between pre-, peri-, and post-natal factors and ASD in preterm births;<br>2. The exposures of interest were pre-, peri-, and post-natal factors in preterm births;<br>3. The outcomes were defined as autism spectrum disorders, autistic disorders, and Asperger syndrome. Outcomes were defined and diagnosed according to self-reports, medical record, or clinical examination;<br>4. Only population-based studies with no geographical or language restrictions.<br>**Exclusion criteria:**<br>1. Intervention studies, review papers, comments, letters, news, notes, protocols, papers or abstracts from conference proceedings.

Supplementary Table 2: Newcastle-Ottawa Scale to assess the quality of included studies

Author, year	Country	Cohort name	Sample size	GA range	Outcome	Selection	Comparability	Exposure	NOS score
Bakian, 2018	USA	Intermountain Healthcare Enterprise Data Warehouse and Utah Registry of Autism and Developmental Disabilities	4,855	22-36 weeks	ASD	4	2	3	9
Buchmayer, 2009	Sweden	Swedish Medical Birth Register and Hospital Discharge Register	502	<37 weeks	Autistic disorder	3	1	2	6
Campbell, 2021	USA	Extremely Low Gestational Age Newborn Study (ELGAN)	858	<28 weeks	ASD	4	2	2	8
Cordero, 2020	USA	The Study to Explore Early Development (SEED)	410	35-37 weeks	ASD	3	2	2	7
Getahun, 2017	USA	Kaiser Permanente Southern California	27,949	28-36 weeks	ASD	4	2	2	8
Ha, 2014	USA	2011 National Survey of Children's Health	9,302	Born at least 3 weeks before reported due date	ASD	2	0	0	2
Hung, 2021	Taiwan	National Health Insurance Research Database	4,468	<37 weeks	ASD	4	2	3	9
Hwang, 2013	Taiwan	National Health Insurance Research Database	30,025	<37 weeks	Infantile autism	4	1	3	8
Jensen, 2022	USA	Extremely Low Gestational Age Newborn Study (ELGAN)	889	<28 weeks	ASD +/- intellectual disability	4	2	3	9
Joseph, 2017	USA	Extremely Low Gestational Age Newborn Study (ELGAN)	840	<28 weeks	ASD +/- intellectual disability	3	0	2	5
Korzeniewski,	USA	Extremely Low Gestational	763	<28 weeks	ASD	3	1	2	6

2018		Age Newborn Study (ELGAN)							
Kuban, 2016	USA	Extremely Low Gestational Age Newborn Study (ELGAN)	874	<28 weeks	ASD	3	2	2	7
Kuzniewicz, 2014	USA	Kaiser Permanente Southern California	3,807	24-34 weeks	ASD	4	2	3	9
Leviton, 2018	USA	Extremely Low Gestational Age Newborn Study (ELGAN)	449	<28 weeks	ASD	3	1	2	6
Logan, 2017	USA	Extremely Low Gestational Age Newborn Study (ELGAN)	874	<28 weeks	ASD	3	1	2	6
Moore, 2012	USA	Office of Statewide Health Planning and Development – merged birth records with maternal and infant discharge records	515,789	23-37 weeks	ASD	4	2	3	9
Toijonen, 2022	Finland	The Finnish Medical Birth Register and the Hospital Discharge Register	23,803	24+0 to 36+6 weeks	ASD	4	2	2	8
Venkatesh, 2020	USA	Extremely Low Gestational Age Newborn Study (ELGAN)	773 - maternal stage/grade; 706 - foetal stage/grade	<28 weeks	ASD	3	1	2	6
Yip, 2017	Norway, Sweden, Denmark, Finland, Western Australia	International Collaboration for Autism Registry Epidemiology (iCARE)	240,042	26-36	ASD	4	1	3	8

GA = gestational age at birth; **ASD** = autism spectrum disorder

Supplementary Table 3: Characteristics of the studies included in the systematic review and meta-analysis.

Author, year	Country	Cohort name	Years of birth	Follow-up period	Preterm sample size	GA range and ascertainment strategy	ASD ascertainment strategy	Examined exposures
Bakian, 2018 ■	USA	Intermountain Healthcare Enterprise Data Warehouse and Utah Registry of Autism and Developmental Disabilities	2002-2010	2004-2014, max 12 years	4,855	22-36 weeks (n/s)	ASD, Medical diagnosis from qualified professional and/or autism special education report	Magnesium level, 5-minute Apgar score, birthweight, race/ethnicity, diabetes, Medicaid insurance status, sex
Buchmayer, 2009	Sweden	Swedish Medical Birth Register and Hospital Discharge Register	1987-2002	Up to age 10, 1987-2005	502	<37 weeks (second-trimester ultrasound findings when possible; otherwise, information on the last menstrual period)	Autistic disorder, Hospital Discharge Register, based on ICD-9 and ICD-10	Hypoglycemia, jaundice, respiratory distress
Campbell, 2021	USA	Extremely Low Gestational Age Newborn Study (ELGAN)	2002-2004	10 years of age	858	<28 weeks (dates of embryo retrieval, intrauterine insemination, or fetal ultrasound before 14th week; when not available, a ≥14 weeks' fetal ultrasound, last menstrual period, or GA log from NICU	ASD, screened with SCQ, then evaluated with ADI-R, and finally administered ADOS-2	Intraventricular hemorrhage, white matter injury

Author, year	Country	Cohort name	Years of birth	Follow-up period	Preterm sample size	GA range and ascertainment strategy	ASD ascertainment strategy	Examined exposures
Cordero, 2020	USA	The Study to Explore Early Development (SEED)	2003-2006	2007-2011	410	35-37 weeks (n/s)	ASD, clinical observation by ADOS and a structured interview administered to caregivers by ADI-R	Neonatal jaundice
Getahun, 2017	USA	Kaiser Permanente Southern California	1991-2009	3-17 years, up to 2013	27,949	28-36 weeks (n/s)	ASD, Physician-diagnosed	Apgar score, placental abruption, preeclampsia, fetal dystocia, malpresentation, prolapsed/nuchal cord, birth asphyxia, neonatal resuscitation
Ha, 2014	USA	2011 National Survey of Children's Health	n/s	One-off survey in 2011	9,302	Born at least 3 weeks before reported due date	ASD, Parental recall of clinician diagnosis	Low birth weight
Hung, 2021	Taiwan	National Health Insurance Research Database	2000-2004	Up to age 18, 2000-2012	4,468	<37 weeks (ICD-9 code(s) not specified)	ASD, Physician-diagnosed	Hyperbilirubinemia in first month of life
Hwang, 2013 ■	Taiwan	National Health Insurance Research Database	1998-2001	Max 11 years, to 2009	30,025	<37 weeks (ICD-9: 765.0, 765.1)	Infantile autism, Physician-diagnosed	Birth weight, intraventricular hemorrhage, hyperbilirubinemia, cerebral dysfunction, hypothyroidism, patent ductus arteriosus, birth asphyxia, bronchopulmonary dysplasia, respiratory distress syndrome, sex

Author, year	Country	Cohort name	Years of birth	Follow-up period	Preterm sample size	GA range and ascertainment strategy	ASD ascertainment strategy	Examined exposures
Jensen, 2022	USA	Extremely Low Gestational Age Newborn Study (ELGAN)	2002-2004	10 years	889	<28 weeks (dates of embryo retrieval, intrauterine insemination, or fetal ultrasound before 14th week; when not available, a ≥14 weeks' fetal ultrasound, last menstrual period, or GA log from NICU	ASD +/- intellectual disability, Questionnaires - SCQ, ADI-R, ADOS-2 followed up by assessment by psychologist	Acid suppressant ≤24 months of age
Joseph, 2017	USA	Extremely Low Gestational Age Newborn Study (ELGAN)	2002-2004	10 years	840	<28 weeks (dates of embryo retrieval, intrauterine insemination, or fetal ultrasound before 14th week; when not available, a ≥14 weeks' fetal ultrasound, last menstrual period, or GA log from NICU	ASD +/- intellectual disability, Questionnaires - SCQ, ADI-R, ADOS-2 followed up by assessment by psychologist	Birth weight, head circumference, cesarean section, race, fever (maternal), infections (maternal, various), magnesium sulfate (neonatal), maternal age, white blood cell count (maternal), pre-pregnancy BMI, SES factors (various), medications (various), obstetric complications (various), singleton, sex, smoking status, years since last pregnancy

Author, year	Country	Cohort name	Years of birth	Follow-up period	Preterm sample size	GA range and ascertainment strategy	ASD ascertainment strategy	Examined exposures
Korzeniewski, 2018	USA	Extremely Low Gestational Age Newborn Study (ELGAN)	2002-2004	10 years	763	<28 weeks (dates of embryo retrieval, intrauterine insemination, or fetal ultrasound before 14th week; when not available, a ≥14 weeks' fetal ultrasound, last menstrual period, or GA log from NICU	ASD, Questionnaires - SCQ, ADI-R, ADOS-2	Inflammation-related protein concentrations in the blood (various)
Kuban, 2016 ■	USA	Extremely Low Gestational Age Newborn Study (ELGAN)	2002-2004	10 years	874	<28 weeks (dates of embryo retrieval, intrauterine insemination, or fetal ultrasound before 14th week; when not available, a ≥14 weeks' fetal ultrasound, last menstrual period, or GA log from NICU	ASD, Questionnaires - SCQ, ADI-R, ADOS-2	Sex

Author, year	Country	Cohort name	Years of birth	Follow-up period	Preterm sample size	GA range and ascertainment strategy	ASD ascertainment strategy	Examined exposures
Kuzniewicz, 2014	USA	Kaiser Permanente Southern California	2000-2007	3-11 years, To 2011	3,807	24-34 (for women with regular menstrual cycles: last menstrual period if in 7-day agreement with a first trimester ultrasound. For women with irregular menstrual cycles: first trimester ultrasound)	ASD, Physician-diagnosed	necrotizing enterocolitis, bacteremia, inotropic support, transfusion, ventilation, intracranial hemorrhage, cystic periventricular leukomalacia, neonatal resuscitation
Leviton, 2018	USA	Extremely Low Gestational Age Newborn Study (ELGAN)	2002-2004	10 years	449	<28 weeks (dates of embryo retrieval, intrauterine insemination, or fetal ultrasound before 14th week; when not available, a ≥14 weeks' fetal ultrasound, last menstrual period, or GA log from NICU	ASD, Questionnaires - SCQ, ADI-R, ADOS-2	Blood levels of IL-4 and IL-10

Author, year	Country	Cohort name	Years of birth	Follow-up period	Preterm sample size	GA range and ascertainment strategy	ASD ascertainment strategy	Examined exposures
Logan, 2017	USA	Extremely Low Gestational Age Newborn Study (ELGAN)	2002-2004	10 years	874	<28 weeks (dates of embryo retrieval, intrauterine insemination, or fetal ultrasound before 14th week; when not available, a ≥14 weeks' fetal ultrasound, last menstrual period, or GA log from NICU	ASD, Questionnaires - SCQ, ADI-R, ADOS-2	SNAP-II (Score for Neonatal Acute Physiology-II) scores
Moore, 2012	USA	Office of Statewide Health Planning and Development – merged birth records with maternal and infant discharge records	1991-2001	Min 5 years, to 2006	515,789	23-37 weeks (n/s)	ASD, Entry for autism in disabilities database	Birth weight for gestational age
Toijonen, 2022	Finland	The Finnish Medical Birth Register and the Hospital Discharge Register	2004-2014	n/s	23,803	24+0 to 36+6 weeks (date of last menstrual period or ultrasound when discrepancy identified)	ASD, ICD-10	Breech delivery

Author, year	Country	Cohort name	Years of birth	Follow-up period	Preterm sample size	GA range and ascertainment strategy	ASD ascertainment strategy	Examined exposures
Venkatesh, 2020	USA	Extremely Low Gestational Age Newborn Study (ELGAN)	2002-2004	10 years	773 for maternal and 706 for fetal stage / grade	<28 weeks (dates of embryo retrieval, intrauterine insemination, or fetal ultrasound before 14th week; when not available, a ≥14 weeks' fetal ultrasound, last menstrual period, or GA log from NICU	ASD, Questionnaires - SCQ, ADI-R, ADOS-2	Histologic chorioamnionitis (maternal and fetal)
Yip, 2017	Norway, Sweden, Denmark, Finland, Western Australia	International Collaboration for Autism Registry Epidemiology (iCARE)	Norway & Sweden 1984-2004, Denmark 1997-2004, Finland 1987-2004, Western Australia 1984-1999	To 2004 in Western Australia, 2006 in Norway, and 2009 in Denmark, Finland, and Sweden	240,042	26-36 weeks (date of last menstrual period or ultrasound when discrepancy identified)	ASD, Medical registries or service/benefits registries	Caesarean section (emergency and planned)

GA = gestational age; **ASD** = autism spectrum disorder; ■ = included in meta-analysis; **n/s** = not specified in article; **SCQ** = Social Communication Questionnaire; **ADI-R** = Autism Diagnostic Interview-Revised; **ADOS-2** = Autism Diagnostic Observation Schedule, Second Edition; **NICU** = neonatal intensive care unit

References

1. Ouzzani M, Hammady H, Fedorowicz Z, Elmagarmid A. Rayyan—a web and mobile app for systematic reviews. Systematic Reviews. 2016;5(1):210.
2. Wells GA, Shea B, O'Connell D, Peterson J, Welch V, Losos M, Tugwell P. The Newcastle–Ottawa Scale (NOS) for Assessing the Quality of Non-Randomized Studies in Meta-Analysis. . Ottawa Hospital Research Institute2000.
